# Genistein chemoprevention of prostate cancer in TRAMP mice

**DOI:** 10.1186/1477-3163-6-3

**Published:** 2007-03-16

**Authors:** Jun Wang, Isam-Eldin Eltoum, Coral A Lamartiniere

**Affiliations:** 1 Department of Pharmacology and Toxicology, University of Alabama at Birmingham, Birmingham, AL, USA; 2 Department of Pathology, University of Alabama at Birmingham, Birmingham, AL, USA; 3 Comprehensive Cancer Center, University of Alabama at Birmingham, Birmingham, AL, USA

## Abstract

Epidemiological studies suggest an inverse association between soy intake and prostate cancer risk. Genistein, the predominant phytoestrogen in soy food, has been proposed as a potential chemopreventive agent due to its anti-estrogen and tyrosine kinase inhibitory effects. To determine the most effective period for genistein chemoprevention, the Transgenic adenocarcinoma mouse prostate (TRAMP) model was used. The treatments were 250 mg genistein/kg AIN-76A diet 1) prepubertally only, 2) in adulthood only or 3) through out life. Controls received AIN-76A diet. By 28 weeks of age, 100% TRAMP mice fed control diet developed prostatic intraepithelial neoplasia (PIN) or adenocarcinomas with 6%, 16%, 44% and 34% developing high grade PIN, well differentiated, moderately differentiated and poorly differentiated prostatic adenocarcinomas, respectively. Prepubertal only (1–35 days postpartum) and adult only genistein treatments (12 – 28 weeks) resulted in 6% and 29% decreases in poorly-differentiated cancerous lesions compared with controls, respectively. The most significant effect was seen in the TRAMP mice exposed to genistein throughout life (1–28 weeks) with a 50% decrease in poorly-differentiated cancerous lesions. In a separate experiment in castrated TRAMP mice, dietary genistein suppressed the development of advanced prostate cancer by 35% compared with controls. Of the tumors that developed in castrated TRAMP mice, 100% were poorly-differentiated in contrast to the 37% of noncastrated TRAMP mice that developed poorly-differentiated tumors. ICI 182,780 (ICI), genistein and estrogen down-regulated androgen receptor (AR), estrogen receptor alpha (ER-α) and progesterone receptor (PR) in the prostates of C57BL/6 mice, and act independently of ER. Our data obtained in intact and castrated transgenic mice suggest that genistein may be a promising chemopreventive agent against androgen-dependent and independent prostate cancers.

## Background

Prostate cancer is the most frequently diagnosed cancer in North American men and accounts for the second largest number of male cancer deaths in the United States [1]. Clinically manifested prostate cancer is less prevalent among Asian men where the intake of soy products is high [2-4]. However, Asians who immigrate to the United States and adopt a Western diet lose this protection [5-8]. High consumption of food containing soy results in high plasma, urine, and prostatic fluid concentrations of phytoestrogens [9,10]. Genistein, the most abundant phytoestrogen component of soy, has been shown to inhibit the growth of both androgen-dependent and independent [11,12] prostate cancer cells. Prostate tumor incidence is significantly reduced in transgenic [13], spontaneous [14], and chemically-induced [15] animal models after ingestion of genistein in the diet at nutritionally relevant concentrations. Mice fed genistein at concentrations of 250 mg/kg AIN-76A diet have serum concentrations at the high physiological level (139 ± 70 nmol/L) [13], comparable with those found in Asian men on a regular soy diet (276 nmol/L) [9,10].

In the past, the study of prostate cancer chemoprevention was hindered by the lack of appropriate animal models. In recent years, genetically manipulated animals have provided new scope for chemoprevention studies and for developing strategies to offset specific genetic susceptibilities to cancer [16,17]. The major advantage of these transgenic models is that cancer arises spontaneously in their natural tissue microenvironment and progresses through multiple stages, as does human cancer. An autochthonous transgenic animal model of prostate cancer, the TRAMP model [18], was developed as a very important tool for understanding the earlier events and the progression of adenocarcinomas. TRAMP mice develop *in situ *and invasive carcinoma of the prostate, mimicking the whole spectrum of human prostate cancer progression from prostatic intra-epithelial neoplasia to large multinodular malignant neoplasia [18-20], as well as androgen-independent disease [21]. Previously, we reported that genistein provided in the diet starting at 5 weeks of age would suppress prostate cancer development in the TRAMP model [13]. In the present study, we investigated 1) timing of exposure to genistein to determine if short term early exposure could exert a permanent protective effect, if adult exposure only would be effective in suppressing prostate tumor development, or if life time genistein exposure was significantly more efficacious, 2) the potential of genistein to suppress androgen-independent prostate cancer, and 3) the dependence of genistein action *via *the estrogen receptor mechanism. Our results demonstrate that genistein in the diet reduces the incidence of poorly differentiated prostatic adenocarcinomas in both intact and castrated TRAMP mice, that the most efficacious protocol is life time exposure, and that genistein action in the prostate occurs *via *the estrogen receptor.

## Materials and methods

### Chemicals

Genistein (98% pure, 1.5% methanol) was a gift from F. Hoffmann-La Roche, Basel, Switzerland. Estradiol benzoate (EB) and dimethyl sulfoxide (DMSO) were purchased from SIGMA, St. Louis, MO. ICI 182,780 was purchased from TOCRIS, Ellisville, MO.

### Animal treatment

All animal studies were conducted in accordance with established guidelines and protocols approved by the UAB Animal Care Committee. Female heterozygous TRAMPs in the background of C57BL/6 were obtained as breeders from the NIH Mouse Models of Human Cancers Consortium. In our studies, TRAMP mice were developed on a pure C57BL/6 background, heterozygous for the probasin-Tag transgene. To produce transgenic offspring, ransgenic females were bred with non-transgenic males because transgenic males develop prostate tumors. After weaning at 3–4 weeks of age, males were separated from females, and a tail biopsy was collected from each mouse. Tail DNA, isolated by standard procedures, was used for determination of transgene incorporation by PCR as described previously [13,22]. Only the males that screened positive for the Tag transgene were used. Male and female breeders were fed standard pellet mouse feed (Harlan Teklad, Madison, WI) until birth of offspring, and then the dams and offspring were switched to AIN-76A diet with or without genistein.

To determine the most effective period of genistein action for chemoprevention 122 intact transgenic males were divided into four treatment groups: 1) controls: transgenic males fed powdered AIN-76A diet from birth until 28 weeks of age, 2) neonatal/prepubertal treatment only: 250 mg genistein/kg AIN-76A diet from birth until five weeks of age, 3) adult exposure only: 250 mg genistein/kg AIN-76A diet from 12 weeks of age until 28 weeks, and 4) lifetime exposure: 250 mg genistein/kg AIN-76A from birth until 28 weeks. AIN-76A diet is a semi-purified diet containing no detectable phytoestrogens. The dietary genistein concentration is the same as the one used in our previous chemoprevention and mechanism of action studies [13,22].

To investigate the effect of genistein treatment on androgen-independent prostate cancer development, TRAMP males fed AIN-76A diet were anesthetized with Ketamine/Xylazine and castrated at 12 weeks of age. After castration, TRAMP males were divided into two groups. Controls were continued on AIN-76A diet (n = 47), whereas the others received 250 mg genistein/kg AIN-76A diet (n = 39) from 12 weeks onward. Animals in both groups were monitored weekly for body weight, tumor progression by abdominal palpation, and survival until 28 weeks of age. Only castrated TRAMP males that developed prostate tumors were used for data evaluation.

To investigate the role of sex steroid receptors and genistein action in the prostate, C57BL/6 mice were used because they were less expensive and labor intensive than TRAMP males. Twelve week old mice were pretreated with 4 μg ICI/g BW 30 min prior to treatment with 500 μg genistein/g BW, 50 ng EB/g BW or an equivalent volume of DMSO (vehicle). The mice were killed six hours after the last treatment and the dorsolateral prostates were dissected for analysis of AR, ER-α and PR. The ICI and genistein concentrations were the same as used in mechanism of action studies in female rats [23]. The EB concentration was shown to be a minimally effective dose to regulate AR, ER-α and PR in the mouse prostate. All injections were subcutaneous. Preliminary experiments were carried out to determine the length of time between genistein and EB treatments to time of measuring the biomarkers. Each group contained 7 samples with 6 dorsolateral prostates each. In our studies, we focused our attention on the dorsolateral prostate, because the dorsolateral lobes of the murine prostate are embryologically homologous to the human prostate [24] where approximately 68% of human prostate cancers originate [25].

### Necropsy and histopatholgy

Necropsy of tumor animals was conducted at 28 weeks of age or when animals became moribund. All major organs were inspected for frank toxicity or evidence of metastases. Evaluations included quantitative and qualitative descriptions of the prostate, lymph nodes, and any tissues showing any visible abnormality. Any tissues containing visible metastases or other abnormalities were also collected for histological evaluation. The urogenital tract, including the bladder, seminal vesicles, prostate and epididymes, were removed, weighed, and prepared for pathological evaluation as previously reported [13]. All tissues were fixed overnight in 10% buffered formalin, and then transferred to 70% ethanol. Fixed tissues were embedded in paraffin, and 5 μm sections were mounted on Colorfrost/Plus microscope slides (Fisher Scientific, Hampton, NH). Sections were stained with hematoxylin and eosin. Histological sections of all tissues were evaluated and reviewed by a board certified pathologist (Dr. I-E. Eltoum) using a scale that has been established for the TRAMP model [26]. Noncancerous lesions were graded as normal tissue, low PIN and high PIN, respectively. Well-, moderately- and poorly-differentiated were used to describe cancerous lesions.

### Immunoblotting

To determine the changes in the expression of AR, ER-α, and PR, prostate tissues were homogenized in RIPA lysis buffer (50 mM Tris-HCl pH 7.4, 1% NP-40, 0.25% Na-deoxycholate, 150 mM NaCl, 1 mM EDTA; Upstate Biotechnology, Charlottesville, Virginia), plus protease inhibitors: 1 μg/ml aprotinin (SIGMA), 1 μg leupeptin (SIGMA), 1 mM PMSF (SIGMA), 1 mM Na3VO4 (SIGMA), and 1 mM NaF (SIGMA). Protein concentration of each sample was determined using the Bradford microtiter plate assay (BioRad, Hercules, CA). The same quantity of protein from each sample was separated by SDS-PAGE and transferred to a nitrocellulose membrane (BioRad, Hercules, CA). The membranes were blocked and immunoblotted with appropriate antibodies including a polyclonal antibody to the amino acid portion of the human AR (N-20) (Santa Cruz, CA); anti-ER-α polyclonal IgG (MC-20) (Santa Cruz); anti-PR (C-19) (Santa Cruz); Molecular weight ladders and reference proteins from the respective companies were used as positive controls. After incubation with HRP-conjugated anti-mouse or anti-rabbit secondary antibody (Pierce, Rockford, IL), protein blots were detected with SuperSignal West Dura Extended Duration Substrate as described by the manufacturer (Pierce, Rockford, IL) and exposed to X-ray radiography film. Quantitative analysis of protein expression was accomplished by scanning autoradiograms and densitometry.

### Data analysis

Statistical analysis of histological specimens used Fisher's exact test to determine significance (P < 0.05). Analyses were conducted using Microsoft Office Excel 2003 (Microsoft Corp., Seattle, WA). For the biochemical data, experiments were analyzed using one way analysis of variance (ANOVA), with subsequent multiple comparisons. The p-values associated with the individual comparisons were completed using separate t-tests.

## Results

### Prostate cancer chemoprevention in intact TRAMPs

A total of 122 TRAMP males were stratified among four different groups according to genistein treatment. Dietary exposure to genistein in the diet at 250 mg/kg diet did not alter body weights at 3, 5 and 28 weeks in all groups (data not shown). Likewise, there were no observed differences in food or water consumption between the control and genistein treated groups. At 28 weeks of age, none of the TRAMP mice on control diet had normal prostates, or low grade PIN (Table 1). Six percent had high grade PIN and 16% had well-differentiated cancerous lesions. In TRAMPs fed control diet, 44% and 34% displayed moderately- and poorly- differentiated carcinomas, respectively. Genistein in the diet from birth until 5 weeks of age only (neonatal/prepubertal exposure), did not alter the development of prostate cancer pathology. However, adult only exposure (weeks 12–28) resulted in a 29% decrease in poorly-differentiated cancerous lesions (34% to 24%). Consistent with this was a 34% increase in moderately differentiated tumors (44% to 59%). The greatest effect was seen in TRAMP exposed to genistein throughout life (1–28 weeks) with a 50% decrease in poorly-differentiated cancerous lesions (34% to 17%). This was accompanied by a 36% increase in moderately differentiated tumors (44% to 60%). Although the percentage of control animals with a score of poorly differentiated tumors was twice that of those fed genistein in the diet for 196 days, there was no statistical difference (Fisher's exact test: p = 0.15). Nevertheless, these data strongly suggest that lifetime, and perhaps adult only, genistein exposure can suppress spontaneously developing prostate cancer in the TRAMP model.

**Table 1:** Effect of genistein on primary pros tate neoplasia in intact TRAMP mice

Genistein (wk)	*n*	PIN 1 (%)	PIN 2 (%)	PIN 3 (%)	WDC (%)	MDC (%)	PDC (%)
None	32	0	0	2 (6)	5 (16)	14 (44)	11 (34)
1–5	31	0	0	2 (6)	7 (23)	12 (39)	10 (32)
12–28	29	0	2 (7)	2 (7)	1 (3)	17 (59)	7 (24)
1–28	30	1 (3)	0	3 (10)	3 (10)	18 (60)	5 (17)

TRAMP mice were sacrificed at 28 weeks of age, and the percentage of mice with normal prostate tissue (PIN 1), low-PIN (PIN 2), high-PIN (PIN 3), well-differentiated carcinoma (WDC), moderately differentiated carcinoma (MDC), and poorly differentiated carcinoma (PDC) was determined.

### Prostate cancer chemoprevention in androgen-independent TRAMP model

In 47 castrated TRAMP mice fed control diet only, 66% were evaluated as having normal prostates while 6% and 3% were characterized as low and high PIN, respectively (Table 2). While no castrated TRAMPs had well differentiated and moderately differentiated adenocarcinomas, 23% displayed poorly differentiated prostate adenocarcinomas. Of those with poorly differentiated adencarcinomas, 27% had lymph node metastases. In animals provided genistein in the diet from time of castration at 12 weeks until necropsy at 28 weeks, the percent of poorly differentiated prostate tumors was decreased by 35% (from 23% to 15%). Likewise, lymph node metastasis (all poorly differentiated) was decreased by 50% (6% to 3%) in genistein-treated mice.

**Table 2:** Effect of genistein on primary prostate neoplasia and lymphatic metastases in castrated TRAMP.

Description	*n*	PIN 1 (%)	PIN 2 (%)	PIN 3 (%)	WDC (%)	MDC (%)	PDC (%)	LNM (%)
Controls	47	31 (66)	3 (6)	2 (4)	0	0	11 (23)	3 (6)
Genistein	39	27 (69)	4 (10)	2 (5)	0	0	6 (15)	1 (3)

TRAMP were castrated at 12 weeks and sacrificed at 28 weeks of age. Percentage of mice with normal prostate tissue (PIN 1), low-PIN (PIN 2), high-PIN (PIN 3), well-differentiated carcinoma (WDC), moderately differentiated carcinoma (MDC), and poorly differentiated carcinoma (PDC) was determined.

### Genistein, estrogen and ICI regulation of steroid receptors in prostates of C57BL/6 mice

**Figure 1:**
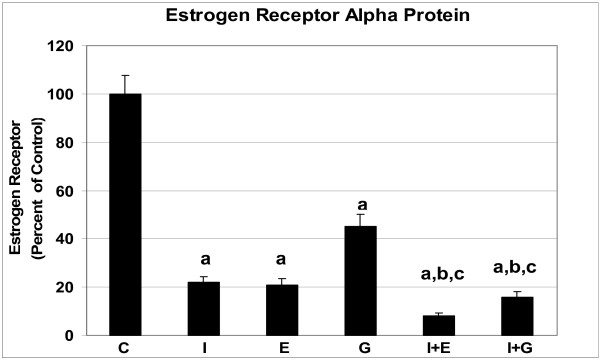
Estrogen receptor expression in dorsolateral prostates of adult mice treated with one injection of vehicle (C), genistein (G) or estradiol benzoate (E) (with or without pretreatment with ICI 182,780 (I) thirty min prior to injection), and sacrificed 16 h later. ^a ^P < 0.001 compared to controls; ^b ^P < 0.001 compared to ICI 182,780 treatment; ^c ^P < 0.001 compared to estrogen or genistein treatments.

**Figure 2:**
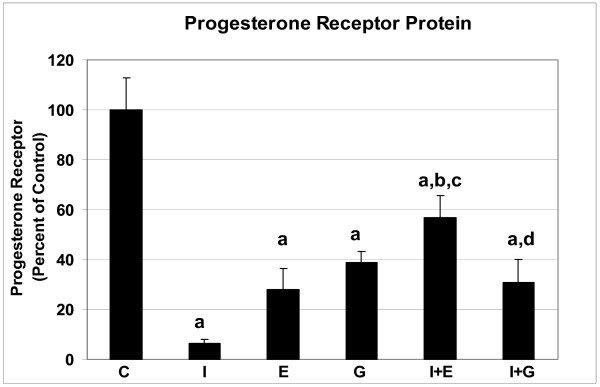
Progesterone receptor expression in dorsolateral prostates of adult mice treated with one injection of vehicle (C), genistein (G) or estradiol benzoate (E) (with or without pretreatment with ICI 182,780 (I) thirty min prior to injection), and sacrificed 16 h later. ^a ^P < 0.001 compared to control; ^b ^P < 0.001 compared to ICI; ^c ^P < 0.05 compared to estrogen treatment; ^d ^P < 0.05 compared to ICI treatment.

**Figure 3:**
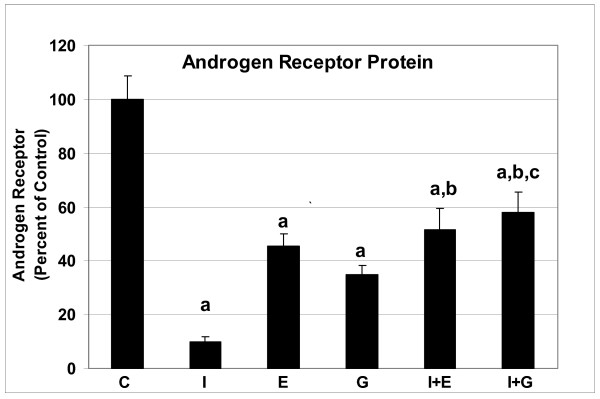
Androgen receptor expression in dorsolateral prostates of adult mice treated with one injection of vehicle (C), genistein (G) or estradiol benzoate (E) (with or without pretreatment with ICI 182,780 (I) thirty min prior to injection), and sacrificed 16 h later. ^a ^P < 0.001 compared to controls; ^b ^P < 0.001 compared to ICI; and ^c ^P < 0.05 compared to genistein treatment.

Since genistein is a phytoestrogen we sought to determine the role of ER in the prostate by blocking ER action with the pure ER antagonist, ICI. We included the use of estradiol benzoate as a positive estrogen treatment. In addition to measuring ER, we measured expression of PR, a protein dependent on ER action [23], and AR since it plays such a prominent role in the prostate. As expected, ICI significantly down-regulated ER-α in the prostate (Figure 1). Likewise, estrogen and genistein had the same effect, albeit genistein was not as effective as estrogen. Furthermore, estrogen and genistein administered with ICI resulted in an additive decrease of ER-α expression. In reference to PR, ICI, estrogen and genistein down-regulated it (Figure 2). However, estrogen administered after ICI resulted in significantly higher PR expression than when estrogen and ICI were administered alone. Genistein administered after ICI resulted in significantly higher PR expression than when ICI was administered alone, but not significantly different from genistein alone. The actions of ICI, estrogen, and genistein administered alone, were able to decrease AR expression in the prostate (Figure 3). In reference to the sequential administration of ICI followed by estrogen, AR protein expression was significantly higher compared to ICI, but not to estrogen treatment. On the other hand, genistein injection after ICI resulted in increased AR compared to single ICI or genistein treatments.

## Discussion

As people live longer, this aging population results in a higher incidence of prostate cancer. Hence, there is need for novel preventive approaches for the management of this disease. Chemoprevention by the use of dietary agents offers a viable option to block the neoplastic inception or delay disease progression. Because prostate cancer is typically diagnosed in men aged 50 years and older, even a slight delay in the onset and subsequent progression of the disease through the use of dietary agents could have important health benefits. Ideally, the efficacy of such chemopreventive agents should be verified in animal models that emulate human disease before recommending their use for humans. In this study, we have used both intact and castrated TRAMP models to evaluate the effect of dietary genistein on prostate cancer development. The advantage of the TRAMP model is that, because of its genetic makeup, initiation of prostate cancer occurs spontaneously. Longitudinal studies in TRAMP mice have shown that over the period of 8–12 weeks of age, this mouse displays progressive stages of prostate cancer found in humans [18-21]. As expected, TRAMP mice produced in our lab developed progressive forms of prostatic disease that resemble human prostate cancer. Careful pathological evaluation revealed that by 28 weeks of age, none of the 32 TRAMP mice on control diet had normal prostates or low PIN. Instead, 94% displayed prostatic adenocarcinomas and 6% had high grade PIN.

For our chemoprevention studies, we focused on the potential of timing of genistein exposure to protect against prostate cancer, a concept that we have shown to be important in genistein suppressing chemically-induced mammary cancer in rats. In the latter, neonatal and prepubertal genistein exposure was necessary to program against mammary cancer [27,28]. However, our prostate cancer studies demonstrated that genistein exposure during the neonatal and prepubertal periods only did not suppress prostate cancer development in TRAMPs. On the other hand, genistein in the diet to adult TRAMP mice resulted in a 29% decrease in poorly-differentiated adenocarcinomas. More effective was life-time genistein treatment. It resulted in a 50% decrease in poorly-differentiated prostate tumors. With both of these genistein treatment groups, the chemoprevention was associated with suppressing the rate of cancer development as evidenced by increased percentage of prostate cancer manifested as moderately differentiated tumors, (44% to 60%). This chemoprevention data is supportive of our earlier studies with genistein action in TRAMPs where genistein in the diet at increasing concentrations starting at 5 weeks suppressed spontaneously developing prostate tumors in a dose-dependent manner [13].

In addition to prostatectomy, radiation and/or chemotherapy, androgen deprivation (castration) has been used as a means of prostate cancer therapy. In most patients the latter leads to reduction in the growth of the primary tumor and its metastases. Unfortunately, the effect lasts only for a year or two. Cancer cells then become androgen independent; they grow (relapse) and eventually kill the patient. Hence, an agent that could prevent or control androgen-independent prostate cancer would have immediate clinical importance. Previously, it was shown that castration of TRAMPs resulted in androgen independent prostate cancer development [21]. Hence, another characteristic of the TRAMP model is its similarity to human disease in the progression to androgen-independent prostate cancer. For our studies, we castrated the mice at 12 weeks, a protocol that is similar to that described by Gingrich et al. [21]. However, we observed only 23% of the TRAMP mice on control diet developing cancerous lesions, with all of them being poorly differentiated prostate tumors. This is to be contrasted to the report of Gingrich et al. that 80% of TRAMP mice developed prostate tumors as early as 12 weeks post castration [21]. Our only explanation for this difference is that they used FVB mice as male background breeders and we used C57BL/6 male mice as background breeders. Apparently, the C57BL/6 background TRAMPs are more resistant to androgen-independent prostate cancer development, thus providing another useful model of prostate cancer study.

Importantly for our study, genistein in the diet suppressed prostate cancer development in these castrated TRAMP mice by 35% and lymph node metastases by 50%. The ability of genistein to reduce poorly differentiated cancer incidence in this model suggests that it may be able to control androgen-independent prostate cancer in man. This is an important manifestation because the latter is a very aggressive and lethal form of cancer. Furthermore, it is most useful and convenient to have the same nutritional agent suppress both androgen-dependent and independent prostate cancers.

Androgen and estrogen play a role in the normal development of the male reproductive tract and are believed to contribute to prostate cancer development [29,30]. Since genistein has been shown to bind to estrogen receptors and to exert estrogen like activity in the female reproductive tract, it has been categorized as a phytoestrogen. However, not all estrogens are exactly the same. Estrogenically-active chemicals are of varying potency and, more importantly, have additional biological actions that can lead to different mechanisms. While genistein action has been extensively investigated in the female, less is known of its actions in the male reproductive tract. For this reason, we investigated the potential of genistein to act *via *the ER mechanism using the potent and pure estrogen antagonist, ICI. Mechanistically, the down-regulation of ER-α protein by ICI is thought to be inhibition of and a change in ER conformation which leads to a rapid loss of ER *via *proteolysis [31,32]. This down regulation of ER is in agreement with our results showing approximately 80% decrease in the ER-α level after ICI treatment. Also, our data demonstrates that ICI was able to down-regulate PR and AR, actions consistent with ICI being a steroid receptor antagonist, even in the prostate.

Studies using cells transfected with ER and PR reporter genes have demonstrated extensive inhibitory cross-reactivity between ER and PR. Both progestins (R5020) and anti-progestins (RU-486) have been shown to act as potent ligand-dependent repressors of ER activity when bound to either isoform of PR [33]. A recent study has pointed to 'reverse' cross-reactivity between these receptors, i.e. blocking of progestin-induced transcription by ICI [34]. Hence, ICI has weak anti-progestational activity [35]. This antiprogestational, in addition to its antiestrogenic, activity of ICI may be important in relation to its anti-cancer activity *in vivo*. In the prostate, AR has been shown to be down-regulated by estrogen during development [29,30]. In our study, AR levels were significantly decreased in the prostate of TRAMPs treated with ICI, suggesting that AR is dependent on ER-α action to be maintained or that ICI has anti-androgen activity.

Interestingly, estrogen and genistein given alone also down-regulated these three sex steroid receptors. Others have shown that ICI is similar to estradiol in its ability to decrease its receptor expression [36,37]. Furthermore, estrogen and genistein given after ICI resulted in additional decrease in ER-α protein in the prostate, suggesting that all three chemicals can act *via *ER. Estrogen and genistein may be decreasing the ER-α *via *feedback/degradation mechanisms rather than directly inhibiting the receptor.

In contrast to ER, PR and AR respond differently to estrogen and genistein given after ICI administration. While ICI down regulated PR and AR, estrogen and genistein given 30 min after ICI resulted in increased PR and AR, partially reversing the ICI effect. This suggests that the effect of ICI on PR and AR is not permanent and that genistein and estrogen do not act exactly like ICI. It has previously been demonstrated that estrogen down regulates ER by feedback/degradation mechanism, but ICI irreversibly and directly inhibits and degrades the ER. It appears that genistein acts similar to estrogen in the prostate. What is not known is whether genistein and estrogen have other mechanisms that make them distinct from each other.

The AR, a member of the steroid receptor family that is activated by testicular androgens, is the major regulatory transcription factor in normal prostate growth and development and in the growth of androgen-dependent prostate cancer. The AR may also contribute to prostate cancer growth during its recurrence in the androgen-deprived patient. A role for AR-mediated gene activation in recurrent prostate cancer is supported by its expression [38,39] together with the expression of androgen-regulated genes [40]. Possible mechanisms for AR reactivation in recurrent prostate cancer include altered growth factor-induced phosphorylation [41-45] and AR mutations [46] that broaden ligand specificity [47]. ER-α, expressed mainly in the stromal compartment of the prostate, may contribute to the pathogenesis of prostate cancer. In a study of the genotypic and allelic frequencies of the six different polymorphic loci of ER-α in a Japanese population, polymorphism in codon 10 of ER-α was found to be a possible risk factor for prostate cancer [48]. Down-regulation of AR and ER-α expression in prostate could be beneficial for chemopreventive activity of genistein. We have previously reported that genistein down-regulated growth factor signaling proteins in the TRAMP model [22]. More specifically, genistein in the diet down-regulated EGF-receptor, IGF-1 receptor and extracellular signaling regulating kinases (ERKs 1 and 2).

The interaction of sex steroid and growth factor signaling pathways is thought to be critical in the process of development and differentiation of hormone-responsive tissues, and for cancer development in the prostate [49]. However, it is unclear whether steroid hormones are mediating the effects of growth factors, or vice versa. Sex steroid-induced epithelial cell proliferation and differentiation have been associated with the coordinated induction of several peptide growth factors and their receptors, including some that are tyrosine kinase dependent. In particular, the EGF- and IGF-signaling pathways are involved in the regulation of cell growth and differentiation. These two protein tyrosine kinase receptors undergo phosphorylation and then utilize their intrinsic kinase activity to phosphorylate downstream mitogen-activated protein kinases, eventually leading to regulation of transcription [reviewed in 50 and 51]. ERK-1 and ERK-2 are particularly important to signal transduction pathways; including nuclear transcription factor regulation, ultimately controlling gene expression. It has been hypothesized that the duration of ERK activity may play a role in control of cellular proliferation and differentiation [52].

It remains to be determined if the action of genistein is directly on these proteins or as a consequence of the AR and ER signaling mechanisms. Estrogen action is strongly related to the EGF and IGF systems with evidence for cross-talk between the systems at several levels [50,51]. The IGF-1R is directly activated by liganded ER, and IGF signaling transcriptionally activates the ER. These growth factor signaling proteins have synergistic effects on cell cycle signaling cascades and proliferation [53]. Genistein has been reported to regulate protein tyrosine kinases [54] of which the EGF- and IGF-1 receptors are examples. Our data points to the actions of genistein in the prostate and the complex regulation of the sex steroid and tyrosine kinase regulated growth factor signaling pathways. Given the similarity of action of genistein and estrogen in the current study, we hypothesize that binding to ER is the primary event of genistein in the prostatic response with biological manifestations for chemoprevention, but without the potent toxic effects of estrogen. In addition to regulating sex steroid- and growth factor-signaling, genistein possess potent anti-oxidant properties [55].

In conclusion, with the androgen dependent model (intact TRAMP), incidences of poorly differentiated prostate tumors were decreased 50% with genistein treatment. Also, we conclude that genistein is effective in suppressing poorly differentiated prostate cancer and metastasis in androgen-independent TRAMP. The mechanisms of genistein chemopreventive activity appears to be through steroid receptor and growth factor signaling pathways.

## Abbreviations

AR: androgen receptor; DMSO: dimethylsulfoxide; EB: estradiol benzoate, ER-α: estrogen receptor-alpha; ERK: extracellular signaling regulating kinase; ICI: ICI 182,780; PIN: prostatic intraepithelial neoplasia; PR: progesterone receptor; TRAMP: transgenic adenocarcinoma mouse prostate

## Authors' contributions

JW carried out the chemoprevention and mechanisms of action studies and drafted the manuscript. I-EE assisted in the necropsy, carried out the histopathology evaluations and assisted in writing the manuscript. CAL proposed the study design and assisted in writing the manuscript.

## References

[B1] Greenlee RT, Murray T, Bolden S, Wingo PA (2000). Cancer statistics, 2000. CA Cancer J Clin.

[B2] Parkin DM, Pisani P, Ferlay J (1999). Global cancer statistics. CA Cancer J Clin.

[B3] Dhom G, Voigt K-D, Knabbe C (1991). Epidemiology of hormone-dependent tumors. Endocrine Dependent Tumors.

[B4] Miller GJ, Coffey DS, Resnick MI, Dorr FA, Karr JP (1998). Diagnosis of stage A prostatic cancer in the People's Republic of China. A Multidisciplinary Analysis of Controversies in the Management of Prostate Cancer.

[B5] Haenszel W, Kurihara M (1968). Studies of Japanese migrants. I. Mortality from cancer and other diseases among Japanese in the United States. J Natl Cancer Inst.

[B6] Shimizu H, Ross RK, Bernstein L, Yatani R, Henderson BE, Mack TM (1991). Cancers of the prostate and breast among Japanese and white immigrants in Los Angeles County. Br J Cancer.

[B7] Whittemore AS, Kolonel LN, Wu AH, John EM, Gallagher RP, Howe GR, Burch JD, Hankin J, Dreon DM, West DW (1995). Prostate cancer in relation to diet, physical activity, and body size in blacks, whites, and Asians in the United States and Canada. J Natl Cancer Inst.

[B8] Cook LS, Goldoft M, Schwartz SM, Weiss NS (1999). Incidence of adenocarcinoma of the prostate in Asian immigrants to the United States and their descendants. J Urol.

[B9] Adlercreutz H, Markkanen H, Watanabe S (1993). Plasma concentrations of phytoestrogens in Japanese men. Lancet.

[B10] Morton MS, Matos-Ferreira A, Abranches-Monteiro L, Correia R, Blacklock N, Chan PS, Cheng C, Chieh-ping W, Griffiths K (1997). Measurement and metabolism of isoflavonoids and lignans in the human male. Cancer Lett.

[B11] Peterson G, Barnes S (1993). Genistein and biochanin A inhibit the growth of human prostate cancer cells but not epidermal growth factor receptor tyrosine autophosphorylation. Prostate.

[B12] Naik HR, Lehr JE, Pienta KJ (1994). An in vitro and in vivo study of antitumor effects of genistein on hormone refractory prostate cancer. Anticancer Res.

[B13] Mentor-Marcel R, Lamartiniere CA, Eltoum I-E, Greenberg NM, Elgavish A (2001). Genistein in the diet reduces the incidence of poorly differentiated prostatic adenocarcinoma in transgenic mice (TRAMP). Cancer Res.

[B14] Pollard M, Wolter W (2000). Prevention of spontaneous prostate-related cancer in lobund wistar rats by a soy protein isolate/isoflavone diet. Prostate.

[B15] Wang J, Eltoum IE, Lamartiniere CA (2002). Dietary genistein suppresses chemically induced prostate cancer in Lobund-Wistar rats. Cancer Lett.

[B16] Alexander J (2000). Use of transgenic mice in identifying chemopreventive agents. Toxicol Lett.

[B17] Hursting SD, Slaga TJ, Fischer SM, DiGiovanni J, Phang JM (1999). Mechanism-based cancer prevention approaches: targets, examples, and the use of transgenic mice. J Natl Cancer Inst.

[B18] Greenberg NM, DeMayo F, Finegold MJ, Medina D, Tilley WD, Aspinall JO, Cunha GR, Donjacour AA, Matusik RJ, Rosen JM (1995). Prostate cancer in a transgenic mouse. Proc Natl Acad Sci USA.

[B19] Gingrich JR, Greenberg NM (1996). A transgenic mouse prostate cancer model. Toxicol Pathol.

[B20] Kaplan-Lefko PJ, Chen TM, Ittmann MM, Barrios RJ, Ayala GE, Huss WJ, Maddison LA, Foster BA, Greenberg NM (2003). Pathobiology of autochthonous CaP in a pre-clinical transgenic mouse model. Prostate.

[B21] Gingrich JR, Barrios Rj, Kattan MW, Nahm HS, Finegold MJ, Greenbierg NM (1997). Androgen-independent prostate cancer progression in the TRAMP model. Cancer Res.

[B22] Wang J, Eltoum I-E, Lamartiniere CA (2004). Genistein regulates growth factor signaling in transgenic mouse model (TRAMP). Molecular and Cellular Endocrinology.

[B23] Cotroneo MS, Fritz WA, Lamartiniere CA (2005). Dynamic profiling of estrogen receptor and epidermal growth factor signaling in the uteri of genistein- and estrogen-treated rats. Food and Chemical Toxicology.

[B24] Price D (1996). Comparative aspects of development and structures in the prostate. Natl Cancer Inst Montogr.

[B25] McNeal JE, Redwine EA, Freiha FS, Stamey TA (1998). Zonal distribution of prostatic adenocarcinoma: correlation with histologic pattern and direction of spread. J Surgical Pathology.

[B26] Wechter WJ, Leipold DD, Murray ED, Quiggle D, McCracken JD, Barrios RS, Greenberg NM (2000). E-7869 (R-flurbiprofen) inhibits progression of prostate cancer in the TRAMP mouse. Cancer Res.

[B27] Fritz WA, Coward L, Wang J, Lamartiniere CA (1998). Dietary genistein: perinatal mammary cancer prevention, bioavailability and toxicity testing in the rat. Carcinogenesis.

[B28] Lamartiniere CA, Cotroneo MS, Fritz WA, Wang J, Mentor-Marcel R, Elgavish A (2002). Genistein chemoprevention: timing and mechanisms of action in murine mammary and prostate. J Nutr.

[B29] Prins GS (1992). Neonatal estrogen exposure induces lobe-specific alterations in adult rat prostate androgen receptor expression. Endocrinology.

[B30] Habenicht UF, el Etreby MF (1988). The periurethral zone of the prostate of the cynomolgus monkey is the most sensitive prostate part for an estrogenic stimulus. Prostate.

[B31] Oliveira CA, Nie R, Carnes K, Franca LR, Prins GS, Saunders PTK, Hess RA (2003). The antiestrogen ICI 182,780 decreases the expression of estrogen receptor-alpha but has no effect on estrogen receptor-beta and androgen receptor in rat efferent ductules. Repro Bio & Endo.

[B32] Wijayaratne AL, Nagel SC, Paige LA, Christensen DJ, Norris JD, Fowlkes DM, McDonnell DP (1999). Comparative analyses of mechanistic differences among antiestrogens. Endocrinology.

[B33] Kraus WL, Weis KE, Katzenellenbogen BS (1995). Inhibitory crosstalk between steroid hormone receptors: differential targeting of estrogen receptor in the repression of its transcriptional activity by agonist- and antagonist-occupied progestin receptors. Mol Cell Biol.

[B34] Nawaz Z, Stancel GM, Hyder SM (1999). The pure antiestrogen ICI 182,780 inhibits progestin- induced transcription. Cancer Res.

[B35] Zand RSR, Grass L, Magklara A, Jenkins DJA, Diamandis EP (2000). Is ICI 182,780 an antiprogestin in addition to being an antiestrogen?. Breast Cancer Research and Treatment.

[B36] Alarid ET, Bakopoulos N, Solodin N (1999). Proteasome-mediated proteolysis of estrogen receptor: a novel component in autologous down-regulation. Mol Endocrinol.

[B37] Preisler-Mashek MT, Solodin N, Stark BL, Tyriver MK, Alarid ET (2002). Ligand- Specific regulation of proteasome-mediated proteolysis of estrogen receptor-alpha. Am J Physiol Endocrinol Metab.

[B38] de Vere White R, Meyers F, Chi SG, Chamberlain S, Siders D, Lee F, Stewart S, Gumerlock PH (1997). Human androgen receptor expression in prostate cancer following androgen ablation. Eur Urol.

[B39] Ruizeveld de Winter JA, Trapman J, Vermey M, Mulder E, Zegers ND, van der Kwast TH (1991). Androgen receptor expression in human tissues: an Immunohistochemical study. J Histochem Cytochem.

[B40] Gregory CW, Hamil KG, Kim D, Hall SH, Pretlow TG, Mohler JL, French FS (1998). Androgen receptor expression in androgen-independent prostate Cancer is associated with increased expression of androgen regulated genes. Cancer Res.

[B41] Culig Z, Hobisch A, Cronauer MV, Radmayr C, Trapman J, Hittmair A, Bartsch G, Klocker H (1994). Androgen receptor activation in prostate tumor cell lines by insulin-like growth factor-I, keratinocyte growth factor, and epidermal growth factor. Cancer Res.

[B42] Nazareth LV, Weigel NL (1996). Activation of the human androgen receptor through a protein kinase A signaling pathway. J Biol Chem.

[B43] Craft N, Shostak Y, Carey M, Sawyers CL (1999). A mechanism for Hormoneindependent prostate cancer through modulation of androgen receptor signaling by the HER-2/neu tyrosine kinase. Nat Med.

[B44] Abreu-Martin MT, Chari A, Palladino AA, Craft NA, Sawyers C (1999). LMitogen- activated protein kinase kinase kinase 1 activates androgen receptor-dependent transcription and apoptosis in prostate cancer. Mol Cell Biol.

[B45] Sadar MD, Hussain M, Bruchovsky N (1999). Prostate cancer: molecular biology of early progression to androgen independence. Endocr Relat Cancer.

[B46] Newmark JR, Hardy DO, Tonb DC, Carter BS, Epstein JI, Isaacs WB, Brown TR, Barrack ER (1992). Androgen receptor gene mutations in human prostate cancer. Proc Natl Acad Sci USA.

[B47] Tan JA, Sharief Y, Hamil KG, Gregory CW, Zang DY, Sar M, Gumerloc H, de Vere White RW, Pretlow TG, Harris SE, Wilson EM, Mohler JL, French FS (1997). Dehydroepiandrosterone activates mutant androgen receptors expressed in the androgen- dependent human prostate cancer xenograft CWR22 and LNCaP cells. Mol Endocrinol.

[B48] Tanaka Y, Sasaki M, Kaneuchi M, Shiina H, Igawa M, Dahiya R (2003). Polymorphisms of estrogen receptor alpha in prostate cancer. Mol Carcinog.

[B49] Cunha GR, Donjacour AA, Cooke PS, Mee S, Bigsby RM, Higgins SJ, Sugimura Y (1987). The endocrinology and development biology of the prostate. Endocrine Reviews.

[B50] Boonstra J, Rijken P, Humbel B, Cremers F, Verkleij A, van Bergen en Henegouwen P (1995). The epidermal growth factor. Cell Biology International.

[B51] Voskuil DW, Vrieling A, van't Veer LJ, Kampman E, Rookus MA (2005). The Insulin- like Growth Factor System in Cancer Prevention. Cancer Epidemiology Biomarkers & Prevention.

[B52] Marshall CJ (1995). Specificity of receptor tyrosine kinase signaling: transient versus sustained extracellular signal regulated kinase activation. Cell.

[B53] Hamelers IH, Steenbergh PH (2003). Interactions between estrogen and insulin-like growth factor signaling pathways in human breast tumor cells. Endocr Relat Cancer.

[B54] Akiyama T, Ishida J, Nakagawa S, Ogawara H, Watanabe S-I, Itho N, Shibuya M, Fukami Y (1987). Genistein, a specific inhibitor of tyrosine-specific protein kinases. J Biol Chem.

[B55] Gyorgy P, Murata K, Ikehata H (1964). Antioxidants isolated from fermented soybeans (tempeh). Nature.

